# On different results for new three step iteration process in Banach spaces

**DOI:** 10.1186/s40064-016-3056-x

**Published:** 2016-09-20

**Authors:** Kifayat Ullah, Muhammad Arshad

**Affiliations:** Department of Mathematics, International Islamic University, Sector H-10, Islamabad, 44000 Pakistan

**Keywords:** Banach space, Iteration process, Stability, Data dependence, Primary 47H09, Secondary 47H10

## Abstract

In this paper we propose a new iteration process, called *AK* iteration process, for approximation of fixed points for contraction mappings. We show that our iteration process is faster than the leading Vatan Two-step iteration process for contraction mappings. Numerical examples are given to support the analytic proofs. Stability of *AK* iteration process and data dependence result for contraction mappings by employing *AK* iteration process are also discussed.

## Introduction and preliminaries

Fixed point theory takes a large amount of literature, since it provides useful tools to solve many problems that have applications in different fields like engineering, economics, chemistry and game theory etc. However, once the existence of a fixed point of some mapping is established, then to find the value of that fixed point is not an easy task that is why we use iteration processes for computing them. By time, many iteration processes have been developed and it is impossible to cover them all. The well-known Banach contraction theorem use Picard iteration process for approximation of fixed point. Some of the other well-known iteration processes are Mann (Mann 1953), Ishikawa (Ishikawa 1974), Agarwal (Agarwal et al. 2007), Noor (Noor 2000), Abbas (Abbas and Nazir 2014), SP (Phuengrattana and Suantai 2011), S$^{*}$ (Karahan and Ozdemir 2013), CR (Chugh et al. 2012), Normal-S (Sahu and Petrusel 2011), Picard Mann (Khan 2013), Picard-S (Gursoy and Karakaya 2014), Thakur New (Thakur et al. 2016) and Vatan Two-step (Karakaya et al. 2015).

Fastness and stability play important role for an iteration process to be preferred on another iteration process. In 1991 , Rhoades mentioned that the Mann iteration process for decreasing function converge faster than the Ishikawa iteration process and for increasing function the Ishikawa iteration process is better than the Mann iteration process. Also the Mann iteration process appears to be independent of the initial guess (see also Rhoades 1977). In Agarwal et al. (2007), the authors claimed that Agarwal iteration process converges at a rate same as that of the Picard iteration process and faster than the Mann iteration process for contraction mappings. In Abbas and Nazir (2014), the authors claimed that Abbas iteration process converge faster than Agarwal iteration process. In Chugh et al. (2012), the authors claimed that CR iteration process is equivalent to and faster than Picard, Mann, Ishikawa, Agarwal, Noor and SP iteration processes for quasi-contractive operators in Banach spaces. Also in Karakaya et al. (2014) the authors proved that CR iteration process converge faster than the S$^{*}$ iterative process for the class of contraction mappings. In Gursoy and Karakaya (2014), authors claimed that Picard-S iteration process converge faster than all Picard, Mann, Ishikawa, Noor, SP, CR, Agarwal, S$^{*},$ Abbas and Normal-S iteration processes for contraction mappings. In Thakur et al. (2016), the authors proved with the help of numerical example that Thakur New iteration process converge faster than Picard, Mann, Ishikawa, Agarwal, Noor and Abbas iteration processes for the class of Suzuki generalized nonexpansive mappings. Similarly, in Karakaya et al. (2015), the authors proved that Vatan Two-step iteration process is faster than Picard-S, CR, SP and Picard-Mann iteration processes for weak contraction mappings. For fragmentation models and processes see Goufo (2014), Goufo and Noutchie (2013). Similarly, for local convergence of Chebyshev–Halley methods with six and eight order of convergence to approximate a locally unique solution of a nonlinear equation see Magrenan and Argyros (2016).

Motivated by above, in this paper, we introduce a new iteration process known as *AK* iteration process and prove analytically that our process is stable. Then we prove that *AK* iteration process converges faster than Vatan Two-step iteration process which is faster than all Picard, Mann, Ishikawa, Noor, SP, CR, S, S$^{*},$ Abbas, Normal-S and Two-step Mann iteration processes for contraction mappings. Numerically we compare the convergence of the *AK* iteration process with the three most leading iteration processes in the existing literature for contraction mappings. The data dependence result for fixed point of contraction mappings by employing *AK* iteration process is also proved.

We now recall some definitions, propositions and lemmas to be used in the next two sections.

A point *p* is called fixed point of a mapping *T* if $T(p)=p$, and *F*(*T*) represents the set of all fixed points of a mapping *T*. Let *C* be a nonempty subset of a Banach space *X*. A mapping $T:C\rightarrow C$ is called contraction if there exists $\theta \in (0,1)$ such that $\left\| Tx-Ty\right\| \le \theta \left\| x-y\right\| ,$ for all $x,y\in C.$

### **Definition 1**

(Berinde 2007) Let $\{a_{n}\}_{n=0}^{\infty }$ and $\{b_{n}\}_{n=0}^{\infty }$ are two real convergent sequences with limits *a* and *b*, respectively. Then we say that $\{a_{n}\}_{n=0}^{\infty }$ converge faster than $\{b_{n}\}_{n=0}^{\infty }$ if

$$\begin{aligned} \lim _{n\rightarrow \infty }\frac{\left\| a_{n}-a\right\| }{\left\| b_{n}-b\right\| }=0. \end{aligned}$$

### **Definition 2**

(Berinde 2007) Let $\{u_{n}\}_{n=0}^{\infty }$ and $\{v_{n}\}_{n=0}^{\infty }$ be two fixed point iteration procedure sequences that converge to the same fixed point *p*. If $\left\| u_{n}-p\right\| \le a_{n}$ and $\left\| v_{n}-p\right\| \le b_{n},$ for all $n\ge 0$, where $\{a_{n}\}_{n=0}^{\infty }$ and $\{b_{n}\}_{n=0}^{\infty }$ are two sequences of positive numbers (converging to zero). Then we say that $\{u_{n}\}_{n=0}^{\infty }$ converge faster than $\{v_{n}\}_{n=0}^{\infty }$ to *p* if $\{a_{n}\}_{n=0}^{\infty }$ converge faster than $\{b_{n}\}_{n=0}^{\infty }$.

### **Definition 3**

(Harder 1987) Let $$\{t_{n}\}_{n=0}^{\infty }$$ be an arbitrary sequence in *C*. Then, an iteration procedure $$x_{n+1}=f(T,x_{n}),$$ converging to fixed point *p*,  is said to be *T*-stable or stable with respect to *T*, if for $$\epsilon _{n}=\left\| t_{n+1}-f(T,t_{n})\right\| ,$$$$n=0,1,2,3, \ldots,$$ we have

$$\begin{aligned} \lim _{n\rightarrow \infty }\epsilon _{n}=0\text { }\Longleftrightarrow \text { }\lim _{n\rightarrow \infty }t_{n}=p. \end{aligned}$$

### **Definition 4**

(Berinde 2007) Let $T,\overset{\sim }{T}:C\rightarrow C$ be two operators. We say that $\overset{\sim }{T}$ is an approximate operator for *T* if, for some $\varepsilon >0$, we have

$$\begin{aligned} \left\| Tx-\overset{\sim }{T}x\right\| \le \varepsilon , \end{aligned}$$

for all $$x\in C$$.

### **Lemma 1**

(Weng 1991) *Let*$\{ \psi _{n}\}_{n=0}^{\infty }$*and*$\{ \varphi _{n}\}_{n=0}^{\infty }$*be nonnegative real sequences satisfying the following inequality:*

$$\begin{aligned} \psi _{n+1}\le (1-\phi _{n})\psi _{n}+\varphi _{n}, \end{aligned}$$

*where*$\phi _{n}\in (0,1)$*for all*$n \in \mathbb {N}$, $\sum \nolimits _{n=0}^{\infty }\phi _{n}=\infty$*and*$\frac{\varphi _{n}}{ \phi _{n}}\rightarrow 0$*as*$n\rightarrow \infty ,$*then*$\lim _{n\rightarrow \infty }\psi _{n}=0.$

### **Lemma 2**

(Soltuz and Grosan 2008) *Let*$\{ \psi _{n}\}_{n=0}^{\infty }$*be nonnegative real sequence for which one assumes there exists*$n_{0}\in \mathbb {N}$*such that for all*$n\ge n_{0},$*the following inequality satisfies:*

$$\begin{aligned} \psi _{n+1}\le (1-\phi _{n})\psi _{n}+\phi _{n}\varphi _{n}, \end{aligned}$$

*where*$\phi _{n}\in (0,1)$*for all*$n\in \mathbb {N} , \sum \nolimits _{n=0}^{\infty }\phi _{n}=\infty$*and*$\varphi _{n}\ge 0$*for all*$n\in \mathbb {N} ,$*then*

$$\begin{aligned} 0\le \underset{n\rightarrow \infty }{\lim \sup }\psi _{n}\le \underset{ n\rightarrow \infty }{\lim \sup \varphi _{n}.} \end{aligned}$$

## AK iteration process and its convergence analysis

Throughout this section we have $$n\ge 0,$$$$\{ \alpha _{n}\}$$ and $$\{ \beta _{n}\}$$ are real sequences in [0, 1], *C* is any subset of Banach space *X* and $$T:C\rightarrow C$$ is any mapping.

 Gursoy and Karakaya (2014) introduced new iteration process called Picard-S iteration process, as follow:

1 $$\begin{aligned} \left\{ \begin{array}{l} u_{0}\in C \\ w_{n}=(1-\beta _{n})u_{n}+\beta _{n}Tu_{n} \\ v_{n}=(1-\alpha _{n})Tu_{n}+\alpha _{n}Tw_{n} \\ u_{n+1}=Tv_{n}. \end{array} \right. \end{aligned}$$

They proved that the Picard-S iteration process can be used to approximate the fixed point of contraction mappings. Also, by providing a numerical example, it is shown that the Picard-S iteration process converge faster than all Picard, Mann, Ishikawa, Noor, SP, CR, S, S$^{*},$ Abbas, Normal-S and Two-step Mann iteration process.

After this Karakaya et al. (2015) introduced a new two step iteration process, we will call it Vatan Two-step iteration process, with the claim that it is even faster than Picard-S iteration process, as follow:

2 $$\begin{aligned} \left\{ \begin{array}{l} u_{0}\in C \\ v_{n}=T((1-\beta _{n})u_{n}+\beta _{n}Tu_{n}) \\ u_{n+1}=T((1-\alpha _{n})v_{n}+\alpha _{n}Tv_{n}). \end{array} \right. \end{aligned}$$

Recently Thakur et al. (2016) used a new iteration process for approximation of fixed points, defined by:

3 $$\begin{aligned} \left\{ \begin{array}{l} u_{0}\in C \\ w_{n}=(1-\beta _{n})u_{n}+\beta _{n}Tu_{n} \\ v_{n}=T((1-\alpha _{n})u_{n}+\alpha _{n}w_{n}) \\ u_{n+1}=Tv_{n}. \end{array} \right. \end{aligned}$$

With the help of numerical example, they proved that their new iteration process is faster than Picard, Mann, Ishikawa, Agarwal, Noor and Abbas iteration processes for some class of mappings. we will call it Thakur New iteration process.

### **Problem 1**

*Is it possible to develop an iteration process whose rate of convergence is even faster than the iteration processes* (1), (2) *and* (3)?

To answer this, we introduce the following new iteration process (4), known as *AK* Iteration Process:

4 $$\begin{aligned} \left\{ \begin{array}{l} x_{0}\in C \\ z_{n}=T((1-\beta _{n})x_{n}+\beta _{n}Tx_{n}) \\ y_{n}=T((1-\alpha _{n})z_{n}+\alpha _{n}Tz_{n}) \\ x_{n+1}=Ty_{n}. \end{array} \right. \end{aligned}$$

We have to prove that our new iteration process (4) is stable and have a good speed of convergence comparatively to other iteration processes.

### **Theorem 1**

*LetCbe a nonempty closed convex subset of a Banach spaceXand*$T:C\rightarrow C$*be a contraction mapping. Let*$\{x_{n}\}_{n=0}^{ \infty }$*be an iterative sequence generated by* (4) *with real sequences*$\{ \alpha _{n}\}_{n=0}^{\infty }$*and*$\{ \beta _{n}\}_{n=0}^{\infty }$*in* [0, 1] *satisfying*$\sum \nolimits _{n=0}^{\infty }\alpha _{n}=\infty$. *Then*$\{x_{n}\}_{n=0}^{\infty }$*converge strongly to a unique fixed point ofT*.

### *Proof*

The well-known Banach theorem guarantees the existence and uniqueness of fixed point *p*. We will show that $x_{n}\rightarrow p$ for $n\rightarrow \infty$. From (4) we have

5 $$\begin{aligned} \left\| z_{n}-p\right\|&= {} \left\| T((1-\beta _{n})x_{n}+\beta _{n}Tx_{n})-p\right\| \nonumber \\&\le {} \theta \left\| (1-\beta _{n})x_{n}+\beta _{n}Tx_{n}-(1-\beta _{n}+\beta _{n})p\right\| \nonumber \\& \le {} \theta ((1-\beta _{n})\left\| x_{n}-p\right\| +\beta _{n}\left\| Tx_{n}-Tp\right\| ) \nonumber \\&\le {} \theta ((1-\beta _{n})\left\| x_{n}-p\right\| +\beta _{n}\theta \left\| x_{n}-p\right\| ) \nonumber \\&= {} \theta (1-\beta _{n}(1-\theta ))\left\| x_{n}-p\right\| . \end{aligned}$$

Similarly,

6 $$\begin{aligned} \left\| y_{n}-p\right\|&= {} \left\| T((1-\alpha _{n})z_{n}+\alpha _{n}Tz_{n})-Tp\right\| \nonumber \\&\le {} \theta \left\| (1-\alpha _{n})z_{n}+\alpha _{n}Tz_{n}-p\right\| \nonumber \\&\le {} \theta [(1-\alpha _{n})\left\| z_{n}-p\right\| +\alpha _{n}\left\| Tz_{n}-p\right\| ] \nonumber \\&\le {} \theta [(1-\alpha _{n})\left\| z_{n}-p\right\| +\alpha _{n}\theta \left\| z_{n}-p\right\| ] \nonumber \\&\le {} \theta (1-\alpha _{n}(1-\theta ))\left\| z_{n}-p\right\| \nonumber \\&\le {} \theta ^{2}(1-\alpha _{n}(1-\theta ))(1-\beta _{n}(1-\theta ))\left\| x_{n}-p\right\| . \end{aligned}$$

Hence

7 $$\begin{aligned} \left\| x_{n+1}-p\right\|&= {} \left\| Ty_{n}-p\right\| \nonumber \\& \le {} \theta \left\| y_{n}-p\right\| \nonumber \\& \le {} \theta ^{3}(1-\alpha _{n}(1-\theta ))(1-\beta _{n}(1-\theta ))\left\| x_{n}-p\right\| \nonumber \\& \le {} \theta ^{3}(1-\alpha _{n}(1-\theta ))\left\| x_{n}-p\right\| , \end{aligned}$$

by using the fact that $(1-\beta _{n}(1-\theta ))<1,$ for $\theta \in (0,1)$ and $\{ \beta _{n}\}_{n=0}^{\infty }$ in [0, 1].

From (7) we have the following inequalities:

8 $$\begin{aligned} \left\{ \begin{array}{l} \left\| x_{n+1}-p\right\| \le \theta ^{3}(1-\alpha _{n}(1-\theta ))\left\| x_{n}-p\right\| ; \\ \left\| x_{n}-p\right\| \le \theta ^{3}(1-\alpha _{n-1}(1-\theta ))\left\| x_{n-1}-p\right\| ; \\ \left\| x_{n-1}-p\right\| \le \theta ^{3}(1-\alpha _{n-2}(1-\theta ))\left\| x_{n-2}-p\right\| ; \\ : \\ : \\ \left\| x_{1}-p\right\| \le \theta ^{3}(1-\alpha _{0}(1-\theta ))\left\| x_{0}-p\right\| . \end{array} \right. \end{aligned}$$

From (8) we can easily derive

9 $$\begin{aligned} \left\| x_{n+1}-p\right\| \le \left\| x_{0}-p\right\| \theta ^{3(n+1)}\prod \limits _{k=0}^{n}(1-\alpha _{k}(1-\theta )), \end{aligned}$$

where $$1-\alpha _{k}(1-\theta )\in (0,1)$$ because $$\theta \in (0,1)$$ and $$\alpha _{n}$$$$\in$$ [0, 1], for all $$n\in \mathbb {N} .$$ Since we know that $$1-x\le e^{-x}$$ for all $$x\in [0,1]$$ ,  so from (9) we get

10 $$\begin{aligned} \left\| x_{n+1}-p\right\| \le \frac{\left\| x_{0}-p\right\| \theta ^{3(n+1)}}{e^{(1-\theta )\sum _{k=0}^{n}\alpha _{k}}}. \end{aligned}$$

Taking the limit of both sides of inequality (10) yields $\lim _{n\rightarrow \infty }\left\| x_{n}-p\right\| =0$, i.e. $x_{n}\rightarrow p$ for $n\rightarrow \infty$, as required. $\square$

### **Theorem 2**

*LetCbe a nonempty closed convex subset of a Banach spaceXand*$T:C\rightarrow C$*be a contraction mapping. Let*$\{x_{n}\}_{n=0}^{\infty }$*be an iterative sequence generated by* (4) *with real sequences*$\{ \alpha _{n}\}_{n=0}^{\infty }$*and*$\{ \beta _{n}\}_{n=0}^{\infty }$*in* [0, 1] *satisfying*$\sum \nolimits _{n=0}^{\infty }\alpha _{n}=\infty$. *Then the iteration process* (4) *isT*-*stable*.

### *Proof*

Let $$\{t_{n}\}_{n=0}^{\infty }$$$$\subset X$$ be any arbitrary sequence in *C*. Let the sequence generated by (4) is $$x_{n+1}=f(T,x_{n})$$ converging to unique fixed point *p* (by Theorem 1) and $$\epsilon _{n}=\left\| t_{n+1}-f(T,t_{n})\right\| .$$ We will prove that $$\lim _{n\rightarrow \infty }\epsilon _{n}=0$$$$\Longleftrightarrow$$$$\lim _{n\rightarrow \infty }t_{n}=p.$$

Let $\lim _{n\rightarrow \infty }\epsilon _{n}=0.$ By using (7) we get

$$\begin{aligned} \left\| t_{n+1}-p\right\|&\le {} \left\| t_{n+1}-f(T,t_{n})\right\| +\left\| f(T,t_{n})-p\right\| \\&= {} \epsilon _{n}+\left\| \begin{array}{l} T(T((1-\alpha _{n})T((1-\beta _{n})t_{n}+\beta _{n}Tt_{n}) \\ +\alpha _{n}T(T((1-\beta _{n})t_{n}+\beta _{n}Tt_{n}))))-p \end{array} \right\| \\&\le {} \theta ^{3}(1-\alpha _{n}(1-\theta ))\left\| t_{n}-p\right\| +\epsilon _{n}. \end{aligned}$$

Define $$\psi _{n}=\left\| t_{n}-p\right\| ,$$$$\phi _{n}=\alpha _{n}(1-\theta )\in (0,1)$$ and $$\varphi _{n}=\epsilon _{n}.$$ Since $$\lim _{n\rightarrow \infty }\epsilon _{n}=0,$$ which implies that $$\frac{ \varphi _{n}}{\phi _{n}}\rightarrow 0$$ as $$n\rightarrow \infty .$$ Thus all conditions of Lemma 1 are fulfilled by above inequality. Hence by Lemma 1 we get $$\lim _{n\rightarrow \infty }t_{n}=p.$$

Conversely let $\lim _{n\rightarrow \infty }t_{n}=p,$ we have

$$\begin{aligned} \epsilon _{n}&= {} \left\| t_{n+1}-f(T,t_{n})\right\| \\&\le {} \left\| t_{n+1}-p\right\| +\left\| f(T,t_{n})-p\right\| \\&\le {} \left\| t_{n+1}-p\right\| +\theta ^{3}(1-\alpha _{n}(1-\theta ))\left\| t_{n}-p\right\| . \end{aligned}$$

This implies that $\lim _{n\rightarrow \infty }\epsilon _{n}=0.$

Hence (4) is stable with respect to *T*. $\square$

### **Theorem 3**

*LetCbe a nonempty closed convex subset of a Banach spaceXand*$T:C\rightarrow C$*be a contraction mapping with fixed pointp*. *Let*$\{u_{n}\}_{n=0}^{\infty }$*and*$\{x_{n}\}_{n=0}^{\infty }$*be an iterative sequences generated by* (2) *and* (4) *respectively, with real sequences*$\{ \alpha _{n}\}_{n=0}^{\infty }$*and*$\{ \beta _{n}\}_{n=0}^{\infty }$*in* [0, 1] *satisfying*$\sum \nolimits _{n=0}^{\infty }\alpha _{n}=\infty$. *Then the following are equivalent:*

(i) *theAKiteration process* (4) *converges to the fixed pointpofT*;
(ii) *the Vatan two-step iteration process* (2) *converges to the fixed pointpofT*.

### *Proof*

First we prove $(i)\Longrightarrow (ii).$ Let the iteration method (4) converges to the fixed point *p* of *T* i.e. $\lim _{n\rightarrow \infty }\left\| x_{n}-p\right\| =0.$ Now using (2) and (4) we have

11 $$\begin{aligned} \left\| z_{n}-v_{n}\right\|&= {} \left\| T((1-\beta _{n})x_{n}+\beta _{n}Tx_{n})-T((1-\beta _{n})u_{n}+\beta _{n}Tu_{n})\right\| \nonumber \\&\le {} \theta \left\{ \left\| (1-\beta _{n})x_{n}+\beta _{n}Tx_{n}-(1-\beta _{n})u_{n}-\beta _{n}Tu_{n}\right\| \right\} \nonumber \\&\le {} \theta \left\{ (1-\beta _{n})\left\| x_{n}-u_{n}\right\| +\beta _{n}\left\| Tx_{n}-Tu_{n}\right\| \right\} \nonumber \\&\le {} \theta \left\{ (1-\beta _{n})\left\| x_{n}-u_{n}\right\| +\theta \beta _{n}\left\| x_{n}-u_{n}\right\| \right\} \nonumber \\&= {} \theta (1-\beta _{n}(1-\theta ))\left\| x_{n}-u_{n}\right\| . \end{aligned}$$

Similarly, using (2) and (4) together with (11) we have

12 $$\begin{aligned} \left\| x_{n+1}-u_{n+1}\right\| &= {} \left\| Ty_{n}-u_{n+1}\right\| \nonumber \\&\le {} \left\| Ty_{n}-y_{n}\right\| +\left\| y_{n}-u_{n+1}\right\| \nonumber \\&= {} \left\| T((1-\alpha _{n})z_{n}+\alpha _{n}Tz_{n})-T((1-\alpha _{n})v_{n}+\alpha _{n}Tv_{n})\right\| \nonumber \\&\quad+\left\| Ty_{n}-y_{n}\right\| \nonumber \\&\le {} \theta \left\| (1-\alpha _{n})z_{n}+\alpha _{n}Tz_{n}-(1-\alpha _{n})v_{n}-\alpha _{n}Tv_{n}\right\| \nonumber \\&\quad+\left\| Ty_{n}-y_{n}\right\| \nonumber \\&\le {} \theta \left\{ (1-\alpha _{n})\left\| z_{n}-v_{n}\right\| +\alpha _{n}\left\| Tz_{n}-Tv_{n}\right\| \right\} +\left\| Ty_{n}-y_{n}\right\| \nonumber \\&\le {} \theta \left\{ (1-\alpha _{n})\left\| z_{n}-v_{n}\right\| +\theta \alpha _{n}\left\| z_{n}-v_{n}\right\| \right\} +\left\| Ty_{n}-y_{n}\right\| \nonumber \\&\le {} \theta (1-\alpha _{n}(1-\theta ))\left\| z_{n}-v_{n}\right\| +\left\| Ty_{n}-y_{n}\right\| \nonumber \\&\le {} \theta ^{2}(1-\alpha _{n}(1-\theta ))(1-\beta _{n}(1-\theta ))\left\| x_{n}-u_{n}\right\| \nonumber \\&\quad+\left\| Ty_{n}-y_{n}\right\| . \end{aligned}$$

For $\{ \beta _{n}\}_{n=0}^{\infty }$ in [0, 1] and $\theta \in (0,1)$, we have

13 $$\begin{aligned} (1-\beta _{n}(1-\theta )<1. \end{aligned}$$

By using (13) together with (12) we get

14 $$\begin{aligned} \left\| x_{n+1}-u_{n+1}\right\| \le (1-\alpha _{n}(1-\theta ))\left\| x_{n}-u_{n}\right\| +\left\| Ty_{n}-y_{n}\right\| . \end{aligned}$$

Define $$\psi _{n}=\left\| x_{n}-u_{n}\right\| ,$$$$\phi _{n}=\alpha _{n}(1-\theta )\in (0,1)$$ and $$\varphi _{n}=\left\| Ty_{n}-y_{n}\right\| .$$

Since $\lim _{n\rightarrow \infty }\left\| x_{n}-p\right\| =0$ and $Tp=p$ so

$$\begin{aligned} \lim _{n\rightarrow \infty }\left\| Ty_{n}-y_{n}\right\| &= {} \lim _{n\rightarrow \infty }\left\| Ty_{n}-Tp+p-y_{n}\right\| \\&\le {} (1+\theta )\lim _{n\rightarrow \infty }\left\| y_{n}-p\right\| \\&= {} 0, \end{aligned}$$

which implies that $\frac{\varphi _{n}}{\phi _{n}}\rightarrow 0$ as $n\rightarrow \infty .$ Thus all conditions of Lemma 1 are fulfilled by (14), so we get

15 $$\begin{aligned} \lim _{n\rightarrow \infty }\psi _{n}=\lim _{n\rightarrow \infty }\left\| x_{n}-u_{n}\right\| =0. \end{aligned}$$

Using (15) we get $\left\| u_{n}-p\right\| \le \left\| x_{n}-u_{n}\right\| +\left\| x_{n}-p\right\| \rightarrow 0$ as $n\rightarrow \infty .$ Hence $\lim _{n\rightarrow \infty }\left\| u_{n}-p\right\| =0$ i.e. the Vatan two-step iteration process (2) converges to the fixed point *p* of *T*.

Next we will prove $(ii)\Longrightarrow (i)$. Let $\lim _{n\rightarrow \infty }\left\| u_{n}-p\right\| =0.$

By using (2) and (4) we have

16 $$\begin{aligned} \left\| v_{n}-z_{n}\right\|&= {} \left\| T((1-\beta _{n})u_{n}+\beta _{n}Tu_{n})-T((1-\beta _{n})x_{n}+\beta _{n}Tx_{n})\right\| \nonumber \\&\le {} \theta \left\{ \left\| (1-\beta _{n})u_{n}+\beta _{n}Tu_{n}-(1-\beta _{n})x_{n}-\beta _{n}Tx_{n}\right\| \right\} \nonumber \\&\le {} \theta \left\{ (1-\beta _{n})\left\| u_{n}-x_{n}\right\| +\beta _{n}\left\| Tu_{n}-Tx_{n}\right\| \right\} \nonumber \\&\le {} \theta \left\{ (1-\beta _{n})\left\| u_{n}-x_{n}\right\| +\theta \beta _{n}\left\| u_{n}-x_{n}\right\| \right\} \nonumber \\&= {} \theta (1-\beta _{n}(1-\theta ))\left\| u_{n}-x_{n}\right\| . \end{aligned}$$

Similarly, using (2) and (4) together with (16) we have

17 $$\begin{aligned} \left\| u_{n+1}-x_{n+1}\right\|&= {} \left\| T((1-\alpha _{n})v_{n}+\alpha _{n}Tv_{n})-Ty_{n}\right\| \nonumber \\&\le {} \theta \left\| (1-\alpha _{n})v_{n}+\alpha _{n}Tv_{n}-y_{n}\right\| \nonumber \\&= {} \theta \left\| (1-\alpha _{n})v_{n}+\alpha _{n}Tv_{n}-(1-\alpha _{n}+\alpha _{n})y_{n}\right\| \nonumber \\&\le {} \theta (1-\alpha _{n})\left\| v_{n}-y_{n}\right\| +\theta \alpha _{n}\left\| Tv_{n}-y_{n}\right\| \nonumber \\&\le {} \theta (1-\alpha _{n})\left\| v_{n}-Tv_{n}\right\| +\theta (1-\alpha _{n})\left\| Tv_{n}-y_{n}\right\| \nonumber \\&\quad+\theta \alpha _{n}\left\| Tv_{n}-y_{n}\right\| \nonumber \\&= {} \theta \left\| Tv_{n}-y_{n}\right\| +\theta (1-\alpha _{n})\left\| v_{n}-Tv_{n}\right\| \nonumber \\&= {} \theta \left\| Tv_{n}-T((1-\alpha _{n})z_{n}+\alpha _{n}Tz_{n})\right\| +\theta (1-\alpha _{n})\left\| v_{n}-Tv_{n}\right\| \nonumber \\&\le {} \theta ^{2}\left\{ (1-\alpha _{n})\left\| v_{n}-z_{n}\right\| +\alpha _{n}\left\| v_{n}-Tz_{n}\right\| \right\} \nonumber \\&\quad+\theta (1-\alpha _{n})\left\| v_{n}-Tv_{n}\right\| \nonumber \\& \le {} \theta ^{2}\left\{ (1-\alpha _{n})\left\| v_{n}-z_{n}\right\| +\alpha _{n}\left\| v_{n}-Tv_{n}\right\| +\alpha _{n}\left\| Tv_{n}-Tz_{n}\right\| \right\} \nonumber \\&\quad+\theta (1-\alpha _{n})\left\| v_{n}-Tv_{n}\right\| \nonumber \\&\le {} \theta ^{2}\left\{ (1-\alpha _{n})\left\| v_{n}-z_{n}\right\| +\theta \alpha _{n}\left\| v_{n}-z_{n}\right\| \right\} \nonumber \\& \quad +\theta ^{2}\alpha _{n}\left\| v_{n}-Tv_{n}\right\| +\theta (1-\alpha _{n})\left\| v_{n}-Tv_{n}\right\| \nonumber \\&= {} \theta ^{2}(1-\alpha _{n}(1-\theta ))\left\| v_{n}-z_{n}\right\| +\theta (1-\alpha _{n}(1-\theta ))\left\| v_{n}-Tv_{n}\right\| \nonumber \\&\le {} \theta ^{3}(1-\alpha _{n}(1-\theta ))(1-\beta _{n}(1-\theta ))\left\| u_{n}-x_{n}\right\| \nonumber \\&\quad+\theta (1-\alpha _{n}(1-\theta ))\left\| v_{n}-Tv_{n}\right\| . \end{aligned}$$

By using (12) together with (17) we get

18 $$\begin{aligned} \left\| u_{n+1}-x_{n+1}\right\| \le (1-\alpha _{n}(1-\theta ))\left\| u_{n}-x_{n}\right\| +(1-\alpha _{n}(1-\theta ))\left\| v_{n}-Tv_{n}\right\| . \end{aligned}$$

Define $$\psi _{n}=\left\| u_{n}-x_{n}\right\| ,$$$$\phi _{n}=\alpha _{n}(1-\theta )\in (0,1)$$ and $$\varphi _{n}=(1-\alpha _{n}(1-\theta ))\left\| v_{n}-Tv_{n}\right\| .$$

Since $\lim _{n\rightarrow \infty }\left\| u_{n}-p\right\| =0$ and $Tp=p,$ so

19 $$\begin{aligned} \lim _{n\rightarrow \infty }\left\| Tv_{n}-v_{n}\right\|&= {} \lim _{n\rightarrow \infty }\left\| Tv_{n}-Tp+p-v_{n}\right\| \nonumber \\&\le {} (1+\theta )\lim _{n\rightarrow \infty }\left\| v_{n}-p\right\| \nonumber \\&= {} 0. \end{aligned}$$

From (19) we have $\frac{\varphi _{n}}{\phi _{n}}\rightarrow 0$ as $n\rightarrow \infty .$ Thus all conditions of Lemma 1 are fulfilled by (18), and so

20 $$\begin{aligned} \lim _{n\rightarrow \infty }\psi _{n}=\lim _{n\rightarrow \infty }\left\| u_{n}-x_{n}\right\| =0. \end{aligned}$$

Using (20) we get $\left\| x_{n}-p\right\| \le \left\| u_{n}-x_{n}\right\| +\left\| u_{n}-p\right\| \rightarrow 0$ as $n\rightarrow \infty .$ Hence $\lim _{n\rightarrow \infty }\left\| x_{n}-p\right\| =0$ i.e. the *AK* iteration process (4) converges to the fixed point *p* of *T*. $\square$

### **Theorem 4**

*LetCbe a nonempty closed convex subset of a Banach spaceXand*$T:C\rightarrow C$*be a contraction mapping with fixed pointp*. *For given*$u_{0}=x_{0}\in C,$*let*$\{u_{n}\}_{n=0}^{\infty }$*and*$\{x_{n}\}_{n=0}^{ \infty }$*be an iterative sequences generated by* (2) *and* (4) *respectively, with real sequences*$\{ \alpha _{n}\}_{n=0}^{\infty }$*and*$\{ \beta _{n}\}_{n=0}^{\infty }$ in [0, 1] *such that*$\alpha \le \alpha _{n}<1,$*for some*$\alpha >0$*and for all*$n\in \mathbb {N}.$*Then*$\{x_{n}\}_{n=0}^{\infty }$*converge topfaster than*$\{u_{n}\}_{n=0}^{\infty }$*does.*

### *Proof*

From (9) we have

21 $$\begin{aligned} \left\| x_{n+1}-p\right\| \le \left\| x_{0}-p\right\| \theta ^{3(n+1)}\prod \limits _{k=0}^{n}(1-\alpha _{k}(1-\theta )). \end{aligned}$$

From iteration process (2), also converging to unique fixed point *p* (Karakaya et al. 2015, Theorem 2) , we have

$$\begin{aligned} \left\| u_{n+1}-p\right\|&= {} \left\| T((1-\alpha _{n})v_{n}+\alpha _{n}Tv_{n})-p\right\| \\&\le {} \theta (1-\alpha _{n}(1-\theta ))\left\| v_{n}-p\right\| \\&= & {} \theta (1-\alpha _{n}(1-\theta ))\left\| T((1-\beta _{n})u_{n}+\beta _{n}Tu_{n})-p\right\| \\&\le {} \theta ^{2}(1-\alpha _{n}(1-\theta ))(1-\beta _{n}(1-\theta ))\left\| u_{n}-p\right\| \\&\le & {} \theta ^{2}(1-\alpha _{n}(1-\theta ))\left\| u_{n}-p\right\| . \end{aligned}$$

Repeating this process n times, we get

22 $$\begin{aligned} \left\| u_{n+1}-p\right\| \le \left\| u_{0}-p\right\| \theta ^{2(n+1)}\prod \limits _{k=0}^{n}(1-\alpha _{k}(1-\theta )). \end{aligned}$$

Since $\alpha \le \alpha _{n}<1$ for some $\alpha >0$ and for all $n\in \mathbb {N},$ so (21) implies that

23 $$\begin{aligned} \left\| x_{n+1}-p\right\|&\le {} \left\| x_{0}-p\right\| \theta ^{3(n+1)}\prod \limits _{k=0}^{n}(1-\alpha (1-\theta )) \nonumber \\&= {} \left\| x_{0}-p\right\| \theta ^{3(n+1)}(1-\alpha (1-\theta ))^{n+1}. \end{aligned}$$

Similarly, (22) together with assumption $\alpha \le \alpha _{n}<1,$ for some $\alpha >0$ and for all $n\in \mathbb {N},$ leads to

24 $$\begin{aligned} \left\| u_{n+1}-p\right\|&\le {} \left\| u_{0}-p\right\| \theta ^{2(n+1)}\prod \limits _{k=0}^{n}(1-\alpha (1-\theta )) \nonumber \\& = {} \left\| u_{0}-p\right\| \theta ^{2(n+1)}(1-\alpha (1-\theta ))^{n+1}. \end{aligned}$$

Define

$$\begin{aligned} a_{n}=\left\| x_{0}-p\right\| \theta ^{3(n+1)}(1-\alpha (1-\theta ))^{n+1}, \end{aligned}$$

and

$$\begin{aligned} b_{n}=\left\| u_{0}-p\right\| \theta ^{2(n+1)}(1-\alpha (1-\theta ))^{n+1}, \end{aligned}$$

then

25 $$\begin{aligned} \Psi _{n}&= {} \frac{a_{n}}{b_{n}} \nonumber \\&= {} \frac{\left\| x_{0}-p\right\| \theta ^{3(n+1)}(1-\alpha (1-\theta ))^{n+1}}{\left\| u_{0}-p\right\| \theta ^{2(n+1)}(1-\alpha (1-\theta ))^{n+1}} \nonumber \\&= {} \theta ^{n+1}. \end{aligned}$$

Since $\lim _{n\rightarrow \infty }\frac{\Psi _{n+1}}{\Psi _{n}} =\lim _{n\rightarrow \infty }\frac{\theta ^{n+2}}{\theta ^{n+1}}=\theta <1,$ so by ratio test $\sum \nolimits _{n=0}^{\infty }\Psi _{n}<\infty .$ Hence from (25) we have,

$$\begin{aligned} \lim _{n\rightarrow \infty }\frac{\left\| x_{n+1}-p\right\| }{\left\| u_{n+1}-p\right\| }=\lim _{n\rightarrow \infty }\frac{a_{n}}{b_{n}} =\lim _{n\rightarrow \infty }\Psi _{n}=0, \end{aligned}$$

which implies that $$\{x_{n}\}_{n=0}^{\infty }$$ is faster than $$\{u_{n}\}_{n=0}^{\infty }.$$$$\square$$

Following are some numerical examples to support analytical proof of Theorem 4 and to illustrate the efficiency of *AK* iteration process (4).

### *Example 1*

Let $T:[0,1]\rightarrow [0,1]$ defined by $T(x)=\frac{x}{2},$ be any mapping. It is easy to see that *T* is a contraction mapping. Hence *T* has a unique fixed point 0.

**Fig. 1:**
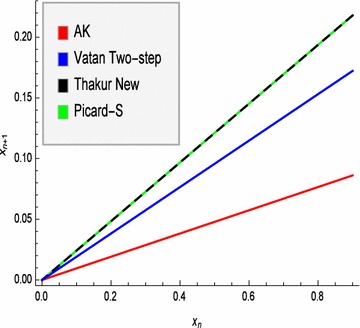
Convergence of AK, Vatan Two-step, Thakur New and Picard-S iterations to the fixed point 0 of mapping $T(x)=\frac{x}{2}$

In Table 1, iterative values generated by our new *AK*, Vatan Two-step, Thakur New and Picard-S iteration processes are given, where $x_{0}=u_{0}=0.9$, $\alpha _{n}=\beta _{n}=\frac{1}{4}$ for all *n* and $n= \overline{0,9}.$ Graphic representation is given in Fig. 1, where sequence of each iteration process for graph is represented by $x_{n}.$ We can easily see that the new *AK* iterations are the first converging one than the Vatan Two-step, the Thakur New and the Picard-S iterations.

**Fig. 2:**
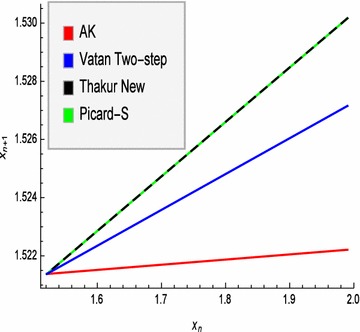
Convergence of AK, Vatan Two-step, Thakur New and Picard-S iterations to the fixed point 1.521379706804568 of mapping $T(x)=(x+2)^{\frac{1}{3}}$

**Table 1:** Iterative values of AK, Vatan Two-step, Thakur New and Picard-S iteration processes for mapping $T(x)=\frac{x}{2},$ where $\alpha _{n}=\beta _{n}=\frac{1}{4},$ for all *n*

	AK	Vatan Two-step	Thakur New	Picard-S
$x_{0}$	0.9	0.9	0.9	0.9
$x_{1}$	$8.6133\times 10^{-2}$	$1.7227\times 10^{-1}$	$2.1797\times 10^{-1}$	$2.1797\times 10^{-1}$
$x_{2}$	$8.2432\times 10^{-3}$	$3.2973\times 10^{-2}$	$5.2789\times 10^{-2}$	$5.2789\times 10^{-2}$
$x_{3}$	$7.889\times 10^{-4}$	$6.3112\times 10^{-3}$	$1.2785\times 10^{-2}$	$1.2785\times 10^{-2}$
$x_{4}$	$7.55\times 10^{-5}$	$1.208\times 10^{-3}$	$3.0963\times 10^{-3}$	$3.0963\times 10^{-3}$
$x_{5}$	$7.2256\times 10^{-6}$	$2.3122\times 10^{-4}$	$7.499\times 10^{-4}$	$7.499\times 10^{-4}$
$x_{6}$	$6.9151\times 10^{-7}$	$4.4257\times 10^{-5}$	$1.8162\times 10^{-4}$	$1.8162\times 10^{-4}$
$x_{7}$	$6.618\times 10^{-8}$	$8.471\times 10^{-6}$	$4.3985\times 10^{-5}$	$4.3985\times 10^{-5}$
$x_{8}$	$6.3336\times 10^{-9}$	$1.6214\times 10^{-6}$	$1.0653\times 10^{-5}$	$1.0653\times 10^{-5}$
$x_{9}$	$6.0615\times 10^{-10}$	$3.1035\times 10^{-7}$	$2.5799\times 10^{-6}$	$2.5799\times 10^{-6}$
$x_{10}$	$5.801\times 10^{-11}$	$5.9402\times 10^{-8}$	$6.2483\times 10^{-7}$	$6.2483\times 10^{-7}$

**Table 2:** Iterative values of AK, Vatan Two-step, Thakur New and Picard-S iteration processes for $\alpha _{n}=\beta _{n}=\frac{1}{4},$ for all *n* and mapping $T(x)=(x+2)^{\frac{1}{3}}$

	AK	Vatan Two-step	Thakur New	Picard-S
$x_{0}$	1.99	1.99	1.99	1.99
$x_{1}$	1.522210596157901	1.527152378405542	1.530163443560674	1.530160376515624
$x_{2}$	1.521381239904628	1.521453635507796	1.521551978236029	1.521551916843118
$x_{3}$	1.521379709633547	1.521380654057891	1.521383088492668	1.521383087287047
$x_{4}$	1.521379706809788	1.521379718941864	1.521379773188262	1.521379773164595
$x_{5}$	1.521379706804577	1.521379706960085	1.521379708107703	1.521379708107238
$x_{6}$	1.521379706804568	1.521379706806560	1.521379706830149	1.521379706830139
$x_{7}$	1.521379706804568	1.521379706804593	1.521379706805070	1.521379706805069
$x_{8}$	1.521379706804568	1.521379706804568	1.521379706804577	1.521379706804577
$x_{9}$	1.521379706804568	1.521379706804568	1.521379706804568	1.521379706804568
$x_{10}$	1.521379706804568	1.521379706804568	1.521379706804568	1.521379706804568
$x_{11}$	1.521379706804568	1.521379706804568	1.521379706804568	1.521379706804568

### *Example 2*

Define a mapping $T:[0,4]\rightarrow [0,4]$ by $T(x)=(x+2)^{\frac{1}{3}}.$ Clearly *T* is a contraction mapping. Hence *T* has a unique fixed point. In Table 2, iterative values generated by our new *AK*, Vatan Two-step, Thakur New and Picard-S iteration processes are given, where $x_{0}=u_{0}=1.99$, $\alpha _{n}=\beta _{n}=\frac{1}{4}$ for all *n* and $n=\overline{0,10}.$

We can easily see thatour new *AK* iterations are the first converging one than the Vatan Two-step, the Thakur New and the Picard-S iterations.

Graphic representation is given in Fig. 2, where sequence of each iteration process is represented by $x_{n}.$

## Data dependence result

Fixed point theory is concerned with investigating a wide variety of issues such as the existence (and uniqueness) of fixed points, the construction of fixed points, etc. One of these themes is data dependency of fixed points. Data dependency of fixed points has been the subject of research in fixed point theory for some time now, and data dependence research is an important theme in its own right. Some work in this direction are Rus and Muresan (1998), Rus et al. (2001, 2003), Berinde (2003), Espínola and Petrusel (2005), Markin (1973), Chifu and Petrusel (2007), Olatinwo (2009, 2010), Soltuz (2001, 2004), Soltuz and Grosan (2008), Chugh and Kumar (2011) and the references therein. In this section, we establish the following data dependence result.

### **Theorem 5**

*Let*$\overset{\sim }{T}$*be an approximate operator of a contraction mappingT*. *Let*$\{x_{n}\}_{n=0}^{\infty }$*be an iterative sequence generated by* (4) *forTand define an iterative sequence*$\{ \overset{\sim }{x} _{n}\}_{n=0}^{\infty }$*as follows*

26 $$\begin{aligned} \left\{ \begin{array}{l} \overset{\sim }{x}_{0}\in C \\ \overset{\sim }{z}_{n}=\overset{\sim }{T}((1-\beta _{n})\overset{\sim }{x} _{n}+\beta _{n}\overset{\sim }{T}\overset{\sim }{x}_{n}) \\ \overset{\sim }{y}_{n}=\overset{\sim }{T}((1-\alpha _{n})\overset{\sim }{z} _{n}+\alpha _{n}\overset{\sim }{T}\overset{\sim }{z}_{n}) \\ \overset{\sim }{x}_{n+1}=\overset{\sim }{T}\overset{\sim }{y}_{n}, \end{array} \right. \end{aligned}$$

*with real sequences*$\{ \alpha _{n}\}_{n=0}^{\infty }$*and*$\{ \beta _{n}\}_{n=0}^{\infty }$*in* [0, 1] *satisfying* (*i*). $\frac{1}{2}\le \alpha _{n},$*for all*$n\in \mathbb {N} ,$*and* (*ii*). $\sum \nolimits _{n=0}^{\infty }\alpha _{n}=\infty .$*If*$Tp=p$*and*$\overset{\sim }{T}\overset{\sim }{p}=\overset{\sim }{p}$*such that*$\lim _{n\rightarrow \infty }\overset{\sim }{x}_{n}=\overset{\sim }{p},$*then we have*

$$\begin{aligned} \left\| p-\overset{\sim }{p}\right\| \le \frac{9\varepsilon }{ 1-\theta }, \end{aligned}$$

*where*$\varepsilon >0$*is a fixed number.*

### *Proof*

It follows from (4) and (26) that

27 $$\begin{aligned} \left\| z_{n}-\overset{\sim }{z}_{n}\right\|&= {} \left\| T((1-\beta _{n})x_{n}+\beta _{n}Tx_{n})-\overset{\sim }{T}((1-\beta _{n}) \overset{\sim }{x}_{n}+\beta _{n}\overset{\sim }{T}\overset{\sim }{x} _{n})\right\| \nonumber \\&\le {} \left\| T((1-\beta _{n})x_{n}+\beta _{n}Tx_{n})-T((1-\beta _{n}) \overset{\sim }{x}_{n}+\beta _{n}\overset{\sim }{T}\overset{\sim }{x} _{n})\right\| \nonumber \\&\quad+\left\| T((1-\beta _{n})\overset{\sim }{x}_{n}+\beta _{n}\overset{ \sim }{T}\overset{\sim }{x}_{n})-\overset{\sim }{T}((1-\beta _{n})\overset{ \sim }{x}_{n}+\beta _{n}\overset{\sim }{T}\overset{\sim }{x}_{n})\right\| \nonumber \\&\le {} \theta \left( (1-\beta _{n})\left\| x_{n}-\overset{\sim }{x} _{n}\right\| +\beta _{n}\left\| Tx_{n}-\overset{\sim }{T}\overset{ \sim }{x}_{n}\right\| \right) +\varepsilon \nonumber \\&\le {} \theta \left( (1-\beta _{n})\left\| x_{n}-\overset{\sim }{x} _{n}\right\| +\beta _{n}\left( \left\| Tx_{n}-T\overset{\sim }{x} _{n}\right\| +\left\| T\overset{\sim }{x}_{n}-\overset{\sim }{T} \overset{\sim }{x}_{n}\right\| \right) \right) \nonumber \\&\quad+\varepsilon \nonumber \\&\le {} \theta (1-\beta _{n}(1-\theta ))\left\| x_{n}-\overset{\sim }{x} _{n}\right\| +\theta \beta _{n}\varepsilon +\varepsilon . \end{aligned}$$

Using (27), we have

28 $$\begin{aligned} \left\| y_{n}-\overset{\sim }{y}_{n}\right\|&= {} \left\| T((1-\alpha _{n})z_{n}+\alpha _{n}Tz_{n})-\overset{\sim }{T}((1-\alpha _{n}) \overset{\sim }{z}_{n}+\alpha _{n}\overset{\sim }{T}\overset{\sim }{z} _{n})\right\| \nonumber \\&\le {} \left\| T((1-\alpha _{n})z_{n}+\alpha _{n}Tz_{n})-T((1-\alpha _{n}) \overset{\sim }{z}_{n}+\alpha _{n}\overset{\sim }{T}\overset{\sim }{z} _{n})\right\| \nonumber \\&\quad+\left\| T((1-\alpha _{n})\overset{\sim }{z}_{n}+\alpha _{n}\overset{ \sim }{T}\overset{\sim }{z}_{n})-\overset{\sim }{T}((1-\alpha _{n})\overset{ \sim }{z}_{n}+\alpha _{n}\overset{\sim }{T}\overset{\sim }{z} _{n})\right\| \nonumber \\&\le {} \theta \left( \left\| (1-\alpha _{n})z_{n}+\alpha _{n}Tz_{n}-(1-\alpha _{n})\overset{\sim }{z}_{n}-\alpha _{n}\overset{\sim }{T }\overset{\sim }{z}_{n}\right\| \right) +\varepsilon \nonumber \\&\le {} \theta \left( (1-\alpha _{n})\left\| z_{n}-\overset{\sim }{z} _{n}\right\| +\alpha _{n}\left\| Tz_{n}-\overset{\sim }{T}\overset{ \sim }{z}_{n}\right\| \right) +\varepsilon \nonumber \\&\le {} \theta \left( (1-\alpha _{n})\left\| z_{n}-\overset{\sim }{z} _{n}\right\| +\alpha _{n}\left( \begin{array}{l} \left\| Tz_{n}-T\overset{\sim }{z}_{n}\right\| \\ +\left\| T\overset{\sim }{z}_{n}-\overset{\sim }{T}\overset{\sim }{z} _{n}\right\| \end{array} \right) \right) +\varepsilon \nonumber \\&\le {} \theta \left( (1-\alpha _{n})\left\| z_{n}-\overset{\sim }{z} _{n}\right\| +\alpha _{n}\left( \theta \left\| z_{n}-\overset{\sim }{z} _{n}\right\| +\varepsilon \right) \right) +\varepsilon \nonumber \\&= {} \theta \left( (1-\alpha _{n}(1-\theta ))\left\| z_{n}-\overset{\sim }{z }_{n}\right\| +\alpha _{n}\varepsilon \right) +\varepsilon \nonumber \\&\le {} \theta \left( (1-\alpha _{n}(1-\theta ))\left( \begin{array}{l} \theta (1-\beta _{n}(1-\theta ))\left\| x_{n}-\overset{\sim }{x} _{n}\right\| \\ +\theta \beta _{n}\varepsilon +\varepsilon \end{array} \right) +\alpha _{n}\varepsilon \right) \nonumber \\&\quad+\varepsilon \nonumber \\&= {} \theta ^{2}(1-\alpha _{n}(1-\theta ))(1-\beta _{n}(1-\theta ))\left\| x_{n}-\overset{\sim }{x}_{n}\right\| +\theta ^{2}\beta _{n}\varepsilon \nonumber \\&-\theta ^{2}\alpha _{n}\beta _{n}\varepsilon +\theta ^{3}\alpha _{n}\beta _{n}\varepsilon +\theta \varepsilon -\theta \alpha _{n}\varepsilon +\theta ^{2}\alpha _{n}\varepsilon +\theta \alpha _{n}\varepsilon +\varepsilon \nonumber \\&= {} \theta ^{2}(1-\alpha _{n}(1-\theta ))(1-\beta _{n}(1-\theta ))\left\| x_{n}-\overset{\sim }{x}_{n}\right\| \nonumber \\&\quad+\theta ^{2}\beta _{n}\varepsilon +\theta ^{2}\alpha _{n}\beta _{n}\varepsilon (\theta -1)+\theta \varepsilon +\theta ^{2}\alpha _{n}\varepsilon +\varepsilon \end{aligned}$$

Similarly, using (28), we have

29 $$\begin{aligned} \left\| x_{n+1}-\overset{\sim }{x}_{n+1}\right\|&= {} \left\| Ty_{n}-\overset{\sim }{T}\overset{\sim }{y}_{n}\right\| \nonumber \\&\le {} \theta \left\| y_{n}-\overset{\sim }{y}_{n}\right\| +\varepsilon \nonumber \\&\le {} \theta ^{3}(1-\alpha _{n}(1-\theta ))(1-\beta _{n}(1-\theta ))\left\| x_{n}-\overset{\sim }{x}_{n}\right\| +\theta ^{3}\beta _{n}\varepsilon \nonumber \\&\quad+\theta ^{3}\alpha _{n}\beta _{n}\varepsilon (\theta -1)+\theta ^{2}\varepsilon +\theta ^{3}\alpha _{n}\varepsilon +\theta \varepsilon +\varepsilon . \end{aligned}$$

For $\{ \alpha _{n}\}_{n=0}^{\infty }$ and $\{ \beta _{n}\}_{n=0}^{\infty }$ in [0, 1] and $\theta \in (0,1)$, we have

30 $$\begin{aligned} \left\{ \begin{array}{l} (1-\beta _{n}(1-\theta )<1 \\ \theta ^{2},\theta ^{3}<1 \\ \theta -1<0 \\ \theta ^{3}\beta _{n}<1, \end{array} \right. \end{aligned}$$

and it follows from assumption (*i*) that

31 $$\begin{aligned} 1-\alpha _{n}<\alpha _{n} \end{aligned}$$

Using (30) and (31) together with (29), we get

32 $$\begin{aligned} \left\| x_{n+1}-\overset{\sim }{x}_{n+1}\right\|&\le {} (1-\alpha _{n}(1-\theta ))\left\| x_{n}-\overset{\sim }{x}_{n}\right\| +\alpha _{n}\varepsilon +4\varepsilon \nonumber \\&= {} (1-\alpha _{n}(1-\theta ))\left\| x_{n}-\overset{\sim }{x}_{n}\right\| +\alpha _{n}\varepsilon +4(1-\alpha _{n}+\alpha _{n})\varepsilon \nonumber \\&\le {} (1-\alpha _{n}(1-\theta ))\left\| x_{n}-\overset{\sim }{x} _{n}\right\| +\alpha _{n}(1-\theta )\frac{9\varepsilon }{1-\theta }. \end{aligned}$$

Let $$\psi _{n}=\left\| x_{n}-\overset{\sim }{x}_{n}\right\| ,$$$$\phi _{n}=\alpha _{n}(1-\theta ),$$$$\varphi _{n}=\frac{9\varepsilon }{1-\theta },$$ then from Lemma 2 together with (32), we get

33 $$\begin{aligned} 0\le \underset{n\rightarrow \infty }{\lim \sup }\left\| x_{n}-\overset{ \sim }{x}_{n}\right\| \le \underset{n\rightarrow \infty }{\lim \sup } \frac{9\varepsilon }{1-\theta }. \end{aligned}$$

Since by Theorem 1 we have $\lim _{n\rightarrow \infty }x_{n}=p$ and by assumption we have $\lim _{n\rightarrow \infty }\overset{\sim }{x}_{n}= \overset{\sim }{p}.$ Using these together with (33), we get

$$\begin{aligned} \left\| p-\overset{\sim }{p}\right\| \le \frac{9\varepsilon }{ 1-\theta }, \end{aligned}$$

as required. $$\square$$

## Conclusions

New iteration process (4) namely *AK* iteration process is introduced for approximating fixed points of contraction mappings. Theorem 1 shows that our new iteration process is also converging to fixed point like other existing iteration processes for contraction mappings. In Theorem 4 we show that our new iteration process is moving faster than the leading Vatan two-step iteration process (2), which was developed by Karakaya et al. (2015). Examples 1 and 2 are given to verify our claim. Our new iteration process is now available for the engineers, computer scientists, physicists as well as mathematicians to solve different problems more efficiently.
